# Supplementary material for: Antipsychotic Medication Influences the Discriminative Value of Acylethanolamides as Biomarkers of Substance Use Disorder
Source: Int J Mol Sci. 2023 May 27;24(11):9371. doi: 10.3390/ijms24119371 (PMC10253203; doi:10.3390/ijms24119371)
Supplement: Supplementary file 1 [file ijms-24-09371-s001.zip › ijms-2269849-supplementary.pdf]

SUPPLEMENTARY MATERIAL

Figures and Tables

Table S1. Plasma concentrations of acylethanolamides (NAEs) in the control, SUD without antipsychotics and SUD + antipsychotics groups.

NAEs	CONTROL (*n* = 175)	SUD (*n* =293)	SUD +Antipsychotic (*n* = 40)	*p* Value
**AEA** median (IQR)	0.33 (0.21-.048)	0.43 (0.30-.061)	0.58 (0.42-0.80)	**0.000**
**DEA** median (IQR)	0.08 (0.05-0.14)	0.12 (0.09-0.17)	0.16 (0.13-0.22)	**0.000**
**DGLEA** median (IQR)	0.07 (0.04-0.09)	0.08 (0.06-0.12)	0.07 (0.11-0.14)	**0.000**
**DHEA** median (IQR)	0.42 (0.27-0.61)	0.52 (0.36-0.70)	0.55 (0.42-0.78)	**0.000**
**LEA** median (IQR)	0.70 (0.51-0.93)	1.07 (0.85-1.37)	1.31 (0.99-1.61)	**0.000**
**OEA** median (IQR)	1.88 (1.33-3.15)	3.32 (2.47-4.47)	3.85 (2.91-5.49)	**0.000**
**PEA** median (IQR)	1.59 (1.27-2.45)	3.37 (2.26-5.65)	3.38 (2.21-6.35)	**0.000**
**POEA** median (IQR)	0.29 (0.16-0.39)	0.30 (0.19-0.42)	0.39 (0.22-0.53)	0.143
**SEA** median (IQR)	4.30 (0.90-5.90)	1.90 (1.24-4.18)	3.01 (1.6-4.36)	0.599

P-value from the Kruskal-Wallis test. *P*-value in bold indicates a statistically significant difference.

Table S2. Logistic regression model for distinguishing patients with SUD from controls.

Logistic regression model								
**Variable**	**B**	**S.E.**	**Wald**	**df**	***p* Value**	**Exp(B)**	**95% CI for EXP(B)**	
							**Lower**	**Upper**
**Sex**	1.209	0.603	4.019	1	0.045	3.349	1.027	10.914
**Age**	-0.017	0.023	0.553	1	0.457	0.983	0.940	1.028
**BMI**	-0.021	0.062	0.121	1	0.728	0.979	0.867	1.104
**AEA[(a)]**	7.708	2.768	7.753	1	0.005	2225.302	9.798	505429.601
**DEA[(a)]**	-3.638	1.735	4.399	1	0.036	0.026	0.001	0.788
**DGLEA[(a)]**	1.456	2.458	0.351	1	0.554	4.289	0.035	530.219
**DHEA[(a)]**	-4.217	1.722	5.996	1	0.014	0.015	0.001	0.431
**LEA[(a)]**	-0.257	2.622	0.010	1	0.922	0.773	0.005	131.766
**OEA[(a)]**	1.346	3.261	0.170	1	0.680	3.842	0.006	2293.625
**PEA[(a)]**	5.256	2.365	4.937	1	0.026	191.634	1.859	19758.811
**POEA[(a)]**	-1.049	1.084	0.937	1	0.333	0.350	0.042	2.931
**SEA[(a)]**	-8.782	2.029	18.737	1	0.000	0.000	0.000	0.008
**Constant**	-0.299	4.286	0.005	1	0.944	0.742		

[(a)]Log10 values
Abbreviations: B = coefficient; BMI = body mass index; CI = confidence interval; df = degrees of freedom; S.E. = standard error; W = Wald test.

Table S3. Logistic regression model for distinguishing patients with SUD and antipsychotic treatment from patients with SUD.

Logistic regression model								
**Variable**	**B**	**S.E.**	**Wald**	**df**	***p* Value**	**Exp(B)**	**95% CI for EXP(B)**	
							**Lower**	**Upper**
**Sex**	0.991	1.220	0.660	1	0.417	2.693	0.247	29.404
**Age**	-0.022	0.030	0.523	1	0.470	0.978	0.922	1.038
**BMI**	0.004	0.062	0.003	1	0.953	1.004	0.890	1.132
**AEA**[(a)]	5.711	4.594	1.546	1	0.214	302.305	0.037	2459340.555
**DEA**[(a)]	-1.161	2.886	0.162	1	0.688	0.313	0.001	89.642
**DGLEA**[(a)]	3.384	2.930	1.334	1	0.248	29.474	0.095	9186.582
**DHEA**[(a)]	0.737	2.113	0.122	1	0.727	2.090	0.033	131.370
**LEA**[(a)]	4.114	2.850	2.083	1	0.149	61.165	0.229	16312.512
**OEA**[(a)]	-6.249	5.344	1.367	1	0.242	0.002	0.000	68.405
**PEA**[(a)]	0.741	3.063	0.059	1	0.809	2.098	0.005	848.644
**POEA**[(a)]	0.017	1.742	0.000	1	0.992	1.017	0.033	30.888
**SEA**[(a)]	-0.504	2.531	0.040	1	0.842	0.604	0.004	86.167
**Constant**	5.689	6.884	0.683	1	0.409	295.575		

[(a)]Log10 values

Abbreviations: B = coefficient; BMI = body mass index; CI = confidence interval; df = degrees of freedom; S.E. = standard error; W = Wald test.

Figure S1

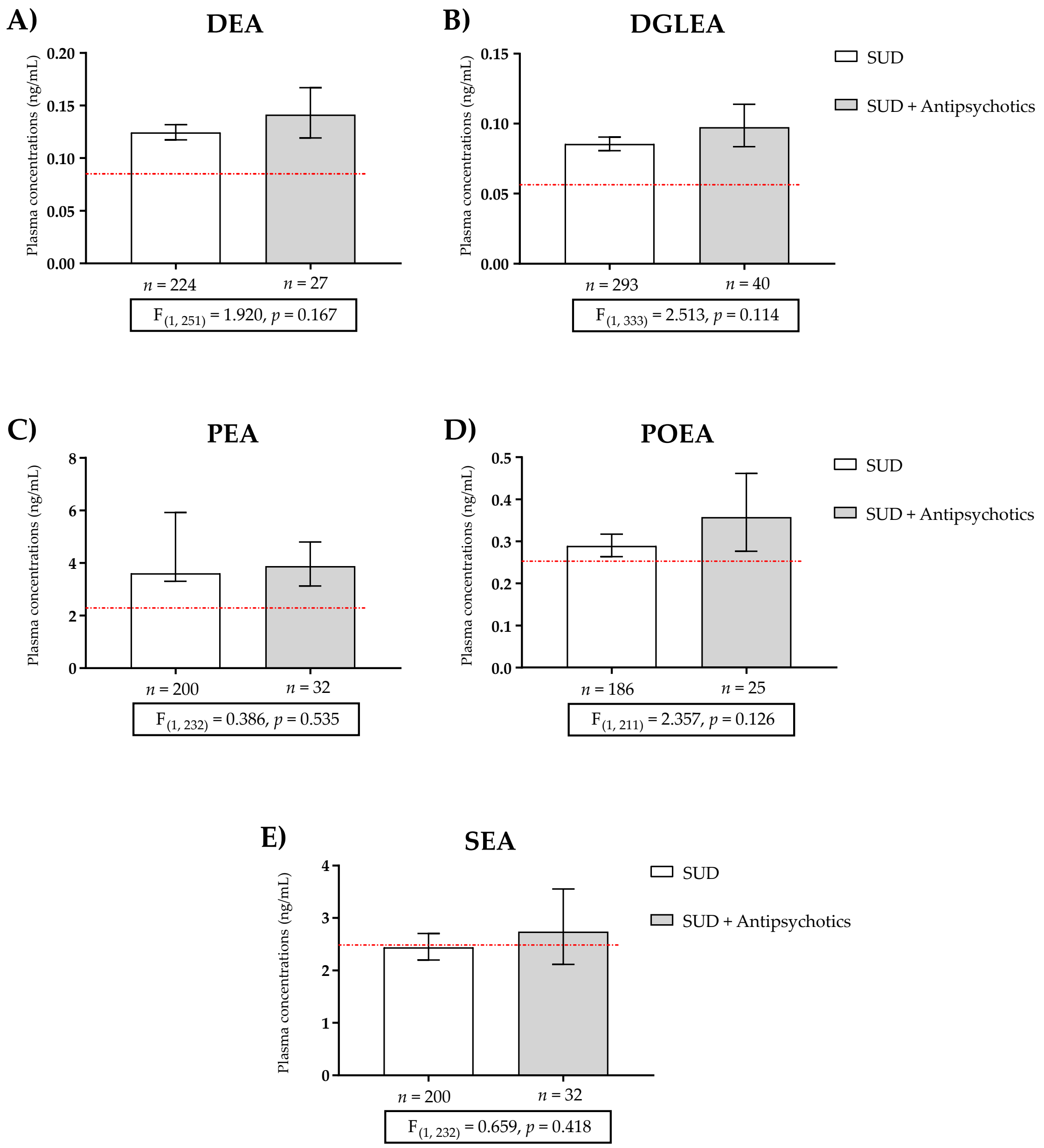

Figure S1. Plasma concentrations of (**A**) docosatetraenoylethanolamide (DEA); (**B**) dihomo-γ-linolenoylethanolamide (DGLEA); (**C**) palmitoylethanolamide (PEA); (**D**) palmitoleoylethanolamide (POEA); and (**E**) stearoylethanolamide (SEA) in patients with substance use disorder (SUD) not using antipsychotics, and patients with SUD using antipsychotics. Red line represents the mean plasma concentration of the control group. Data were analyzed by one-way analysis of covariance (ANCOVA). Bars are the estimated marginal means and 95% confidence intervals (CI).

Figure S2

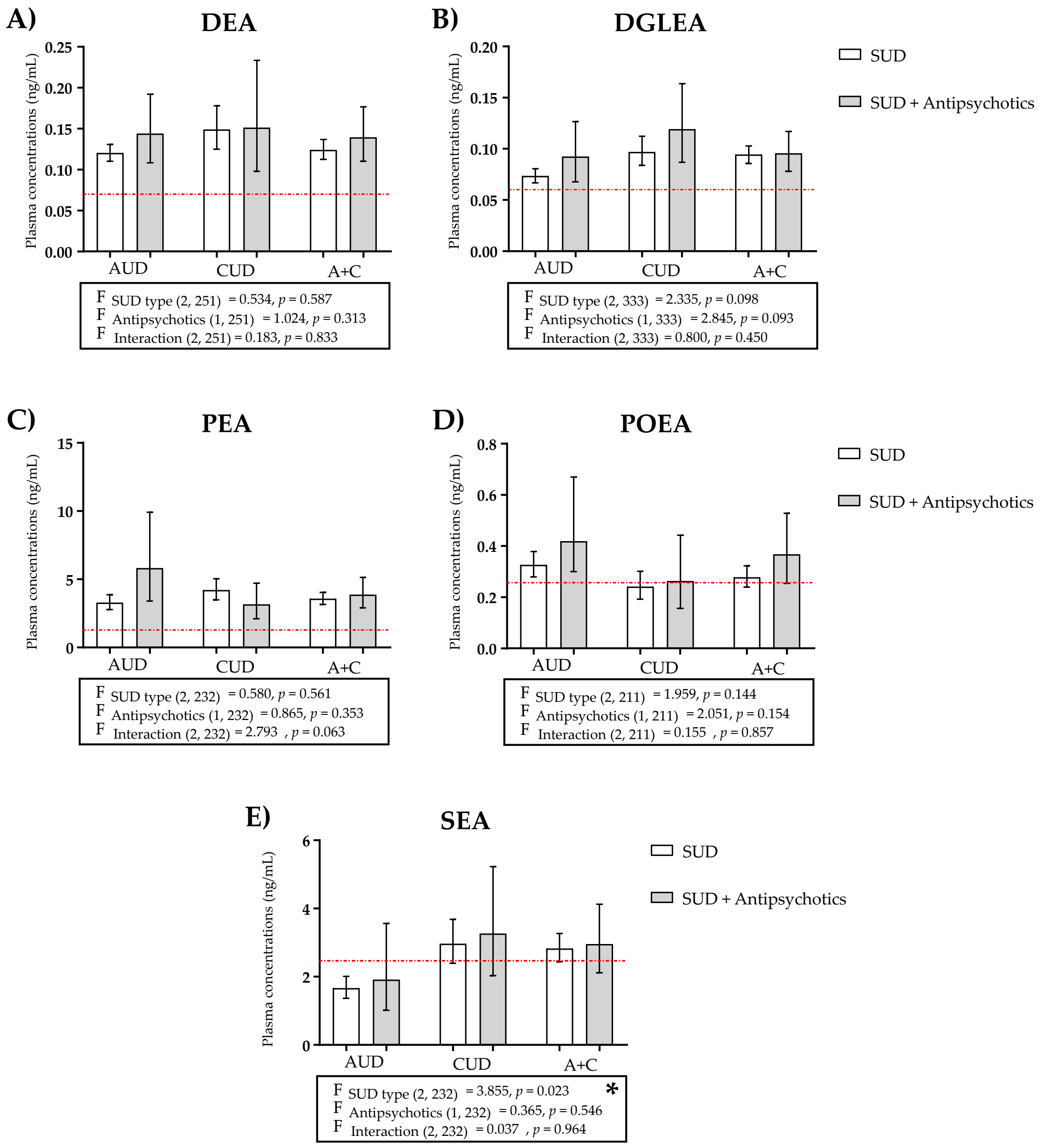

Figure S2. Plasma concentrations of (**A)** DEA; (**B)** DGLEA; (**C)** PEA; (**D)** POEA; and (**E**) SEA in patients with SUD not using antipsychotics and patients of SUD using antipsychotics classified on the basis of AUD, CUD or AUD + CUD diagnosis. Red line represents the mean plasma concentration of the control group. Data were analyzed by two-way ANCOVA. Bars are estimated marginal means and 95% CI. (*) $p < 0.05$ for factors (F).
